# Effect of non-invasive transcutaneous auricular vagus nerve stimulation (taVNS) on non-motor symptoms in multiple sclerosis: study protocol for a randomised, controlled trial

**DOI:** 10.1136/bmjopen-2025-103998

**Published:** 2025-11-09

**Authors:** Thorsten Herr, Julie Gamain, Sebastian Strauss, Christoph Szeska, Agnes Flöel, Iris-Katharina Penner, Mathias Weymar, Matthias Grothe

**Affiliations:** 1 Department of Neurology, University Medicine Greifswald, Greifswald, Germany; 2 Department for Radiology, University Medicine Greifswald, Greifswald, Germany; 3 Department of Biological Psychology and Affective Science, University of Potsdam, Potsdam, Germany; 4 Department of Neurology, German Centre for Neurodegenerative Diseases, Bonn, Germany; 5 Inselspital University Hospital Bern, Bern, Switzerland

**Keywords:** Multiple sclerosis, NEUROPHYSIOLOGY, Neurophysiology

## Abstract

**Introduction:**

Research in people with relapsing remitting multiple sclerosis (PwRRMS) is increasingly focusing on non-motor symptoms like cognitive impairment, fatigue and depression. Due to the high negative impact on quality of life and high socioeconomic costs based on these symptoms, more specific research to improve non-motor symptoms is needed. Transcutaneous auricular vagus nerve stimulation (taVNS) has been found to be a cognitive enhancer in preclinical research and was successfully used for the treatment of psychiatric and neurological disorders to combat dysfunctional cognitive and affective processes. However, the capacity of taVNS to improve cognitive and other non-motor symptoms in PwRRMS has not been tested yet. The aim of this study is to evaluate the therapeutic potential of taVNS on cognitive processing speed. Based on ample evidence demonstrating that taVNS promotes adaptive cognitive and affective processes, we hypothesised that taVNS would alleviate cognitive processing speed in PwRRMS.

**Methods and analysis:**

This study protocol describes the prospective, single-centre, SHAM-controlled, single-blinded trial with a planned sample size of 60 participants (30 PwRRMS, with a diagnosis of multiple sclerosis according to McDonald criteria and 30 healthy controls; age: 18–50 years). The Symbol Digit Modalities Test (SDMT) will be used to determine cognitive processing speed, Beck Depression Inventory-II to determine depression and Fatigue Scale for Motor and Cognitive Functions to determine fatigue. The severity of multiple sclerosis will be assessed using the Expanded Disability Status Scale. After baseline assessment, a taVNS protocol (duration: 30 min, tolerance threshold, pulse width: 250 μs, stimulation frequency: 25 Hz, 30 s on/30 s off) will be applied, followed by post-intervention assessment.

**Ethics and dissemination:**

The study was reviewed and approved by the local ethics committee of the University Medical Centre Greifswald (study reference number: BB137/24). Clinical trial registration: www.drks.de, number: DRKS00034912. Study results will be disseminated through academic conferences as well as peer-reviewed publications.

**Trial registration number:**

DRKS00034912.

**STRENGTHS AND LIMITATIONS OF THIS STUDY**

- This is the first study assessing the effect of a non-invasive transcutaneous auricular vagus nerve stimulation intervention on non-motor symptoms in people with multiple sclerosis.
- Due to the single centre setting and the planned sample size, statistical power to detect possible smaller effect sizes as well as the external validity of this study might be limited.
- Verum and SHAM interventions are included in this study, and the primary and secondary outcomes are clearly defined.
- SHAM intervention is performed at the earlobe which is in very rare cases innervated by vagal nerve fibres.
- Well-established tests and questionnaires are used for the neuropsychological assessment like the Symbol Digit Modalities Test, the Beck Depression Inventory-II and the Fatigue Scale for Motor and Cognitive Functions.

## Introduction

 Relapsing remitting multiple sclerosis (RRMS) is one of the most prevalent neurological disorders, especially in young adults.^1^ The disease can lead to many different neurological signs and symptoms, such as paresis or hypoaesthesia, as well as cognitive impairment occurring in 40%–70% of people with RRMS (PwRRMS).^2^ The most prevalent impaired cognitive domain is processing speed, which has a significant impact on quality of life.^3^ While highly effective immunomodulatory drugs are available to suppress disease activity, there are no specific treatment options for cognitive impairment.

A new therapeutic approach might be transcutaneous auricular vagus nerve stimulation (taVNS), which is a safe, non-invasive method that stimulates the auricular branch of the vagus nerve.^4^,^7^ Stimulation of the auricular branch of the vagus nerve leads to an activation of the nucleus tractus solitarius (NTS), which, in turn, leads to an activation or modulation of different cortical and subcortical brain regions, including the serotoninergic raphe nuclei, the cholinergic nucleus basalis of Meynert, the pedunculopontine nucleus and the noradrenergic locus caeruleus.^8^,^13^ Accordingly, taVNS leads to a widespread modulation of different neurotransmitter systems, including the serotonergic, cholinergic and noradrenergic, but also the GABAergic system.^10 14 15^ The effects of the taVNS on these neurotransmitter systems have been demonstrated in various animal experiments.^16^,^20^ Building on these studies, several assessments were carried out on healthy volunteers, which used functional MRI(fMRI) to demonstrate the modulation of the above-mentioned neurotransmitter systems due to taVNS.^14 21^ The structures modified by taVNS, include, among others, the locus caeruleus and the NTS, but also cortical areas such as the postcentral gyrus, the cerebellum, the prefrontal cortex, the insula and the anterior cingulate.^14 21^ The interplay of these neural circuits has been shown to underlie cognitive and affective processes, such as executive function.^22^

Recent studies provide evidence for taVNS to express beneficial effects on cognitive outcomes in healthy adults, showing improvements, for example, in spatial working memory, executive functions, learning or motivation after taVNS compared with control conditions.^23^,^30^ Furthermore, there are first results showing an improvement in cognition when long-term stimulation (n=76; 2-week taVNS, daily use for 4 hours/day) is used in healthy adults.^31^

Moreover, the effect of taVNS on cognition has also been assessed in neurological conditions, including epilepsy, stroke and mild cognitive impairment due to neurodegenerative disorders.^32^,^34^

Until now, the effect of taVNS on processing speed in PwRRMS has never been assessed. Based on the already existing promising effects on cognitive domains in other diseases due to neurotransmitter changes and the cortical and subcortical alterations due to the neurotransmitter modification, taVNS might have the potential to alter processing speed in PwRRMS.

In 2020, Grothe *et al* revealed the functional representation of the Symbol Digit Modalities Test (SDMT) in PwRRMS. The results showed an activation of frontal, occipital, parietal and cerebellar areas under performance of the SDMT.^35^ The neuroanatomical overlap between cortical areas that are activated due to taVNS and the areas that are crucial for processing speed performance (cerebellum and prefrontal cortex) might support the hypothesis that taVNS can alter processing speed performance.^14 21 35^

Based on the neurotransmitter network alterations and the overlap between the cortical and subcortical areas that are affected by taVNS and are responsible for processing speed performance in PwRRMS, the aim of this study is to assess the effect of taVNS on processing speed in PwRRMS (primary). Furthermore, the association between disease-related variables (disease severity, fatigue and depression) and the change in processing speed will be evaluated (secondary). This protocol is intended to describe the background, planned methods and procedures for the proposed study.

## Methods and analysis

### Study design

This is a prospective, single-centre, single-blinded, SHAM-controlled trial to evaluate the effect of taVNS on non-motor symptoms in PwRRMS. Study begin will be 1 May 2025. The study was reviewed and approved by the local ethics committee of the University Medical Center Greifswald (study reference number: BB137/24). Clinical trial registration: www.drks.de, trial registration number: DRKS00034912.

### Study population and recruitment

In total, 60 subjects will be included in this study, 30 PwRRMS and 30 healthy controls. PwRRMS will be recruited from the MS outpatient clinic of the University Medicine Greifswald. The healthy control group will be recruited through advertisement. Every participant will sign informed consent. An example of the participant consent form is added as online supplemental material.

#### Patient and public Involvement

Patients as well as the public were not involved in the study design, conduct, reporting or dissemination plans of this research.

### Inclusion/exclusion criteria

#### Inclusion criteria

Participants will be eligible for the study according to the following criteria: (1) age: 18–50 years; (2) able to agree to participate and sign informed consent; and (3) diagnosed with RRMS according to the McDonald criteria 2017 (patient group).^36^

#### Exclusion criteria

Exclusion criteria are: (1) history or current acute or chronic illness (eg, dermatological diseases because of a potential irritation and worsening due to a stimulation at the earlobe or specific cardiovascular diseases (such as bradycardia, myocardial infarction and heart failure) as taVNS potentially leads to a modulation of heart rate variability which, in turn, might have a negative impact on cardiac pathologies)^37^; (2) presence of other neurological diseases (except for RRMS in the patient group) or psychiatric diseases; (3) history of medication (except immunomodulatory medication in RRMS as well as symptomatic treatment, including antispastic medication); (4) pregnancy; and (5) participation in other, possibly conflicting studies.

### Interventions

#### Neuropsychological evaluation

##### Symbol Digit Modalities Test

Cognitive processing speed will be assessed using the SDMT.^38^,^40^ The SDMT comprises two parts: a legend with the numbers 1 through 9 with corresponding, meaningless symbols for each number (in total nine different symbols) as well as several lines with symbols in a random order and a space under each symbol. The subjects have to complete as many lines in 90 s as possible (telling the correct number under the corresponding symbol according to the legend at the top of the page). For the planned study, the total score, which is defined as the total amount of correct matched symbol–number pairs, will be taken into account for analysis.^41^ Different SDMT versions will be used to control multiple testing and to avoid learning effects.

##### Beck Depression Inventory-II Test

Depression will be quantified with the Beck Depression Inventory-II Test (BDI-II; Hogrefe Austria GmbH, Wien, Austria).^42^,^45^ The test consists of 21 groups of statements with corresponding points scaling from 0 to 3 towards a specific topic. The participants can choose only one statement for each topic. The total score (sum of the point values of all chosen statements) indicates the presence of depression as well as a graduation between mild, moderate and severe depression.^43 44^

##### Fatigue Scale for Motor and Cognitive Functions

The Fatigue Scale for Motor and Cognitive Functions (FSMC) is a screening tool with a high sensitivity as well as a high specificity to detect trait fatigue and furthermore differentiates between motor and cognitive fatigue.^46^ The FSMC consists of 20 items with 1–5 points for each item (1=‘does not apply at all’ to 5=‘fully applies’; 1–5 points).^46^ The total score as well as the sub-scores for cognitive and motor fatigue will be analysed in the planned study.

##### Expanded Disability Status Scale

The Expanded Disability Status Scale (EDSS) will be performed to evaluate the degree of disability in PwRRMS. The scale ranges from 0 to 10 and is primarily focused on motor skills.^47^ A score of 0 describes normal clinical findings whereas a score of 10 means death due to multiple sclerosis.^47 48^

### taVNS administration

Either SHAM stimulation or verum stimulation will be performed via a taVNS-R device (taVNS Technologies GmbH, Erlangen, Germany). This device consists of two iridium-covered, round titanium electrodes of 2 mm diameter, applying a small electric current to the skin. The device will be attached to the left ear. For verum stimulation, the electrodes will be applied to the cymba conchae; for SHAM stimulation, the electrodes will be applied to the left-sided earlobe of the subject. Before the stimulation, the ear of the subject will be prepared using an alcohol wipe for cleaning the skin from, for example, particles. Once attached to the ear of the subject, the stimulation intensity will be individually calibrated to ensure that intensity will be at tolerance threshold (defined as a painless but strong tingling sensation). For this, the applied current will be increased stepwise (100 μA steps) until a painful sensation is reported by the subject. The stimulation current will be noted and the current will be reduced until the subjects feel a strong tingling sensation without pain perception (this current will also be noted). This procedure will be repeated twice and the perception threshold will be calculated as the mean value of the four tested current values.^29^

The stimulation will be delivered continuously 15 min before, during, as well as 5 min after the neuropsychological testing (SDMT) regardless of SHAM stimulation or verum stimulation. The following stimulation parameters will be applied: pulse width: 250 μs, stimulation frequency: 25 Hz, 30 s on/30 s off, motion threshold: off, ramp-up: off, buzzer: off. Stimulation settings will be controlled by a taVNS Research App (taVNS Technologies) via bluetooth connection with a mobile phone. In order to control for circadian influences within subjects, stimulation will be applied at a constant time for each participant (when the participant will be assessed in the morning, the second assessment will also be performed in the morning).

#### Adverse effects

taVNS is, as already described, a non-invasive method to stimulate the auricular branch of the vagal nerve.^5^,^7^ In 2023, Tan *et al* published a systematic review and meta-analysis showing the safety of taVNS application,^4^ see also pooled data analysis by Giraudier *et al.*^5^ In the case of the occurrence of any adverse events during the planned study (for example, experience of pain, itching of the skin, skin irritation, dizziness, etc), a corresponding report will be created by the research assistant. The adverse event will be evaluated towards a potential causal relationship to the treatment and the severity of the adverse event.

### Randomisation

Subjects will participate in two study sessions (day 1 and day 2), in which the participants will receive either SHAM stimulation or verum stimulation (cross-over design; stimulation will be counterbalanced across sessions). The respective stimulation condition in the first session will be determined using a lottery procedure (online randomisation tool). In summary, each participant will switch from verum to SHAM and vice versa.

### Study schedule

#### Intervention groups

**Figure 1:**
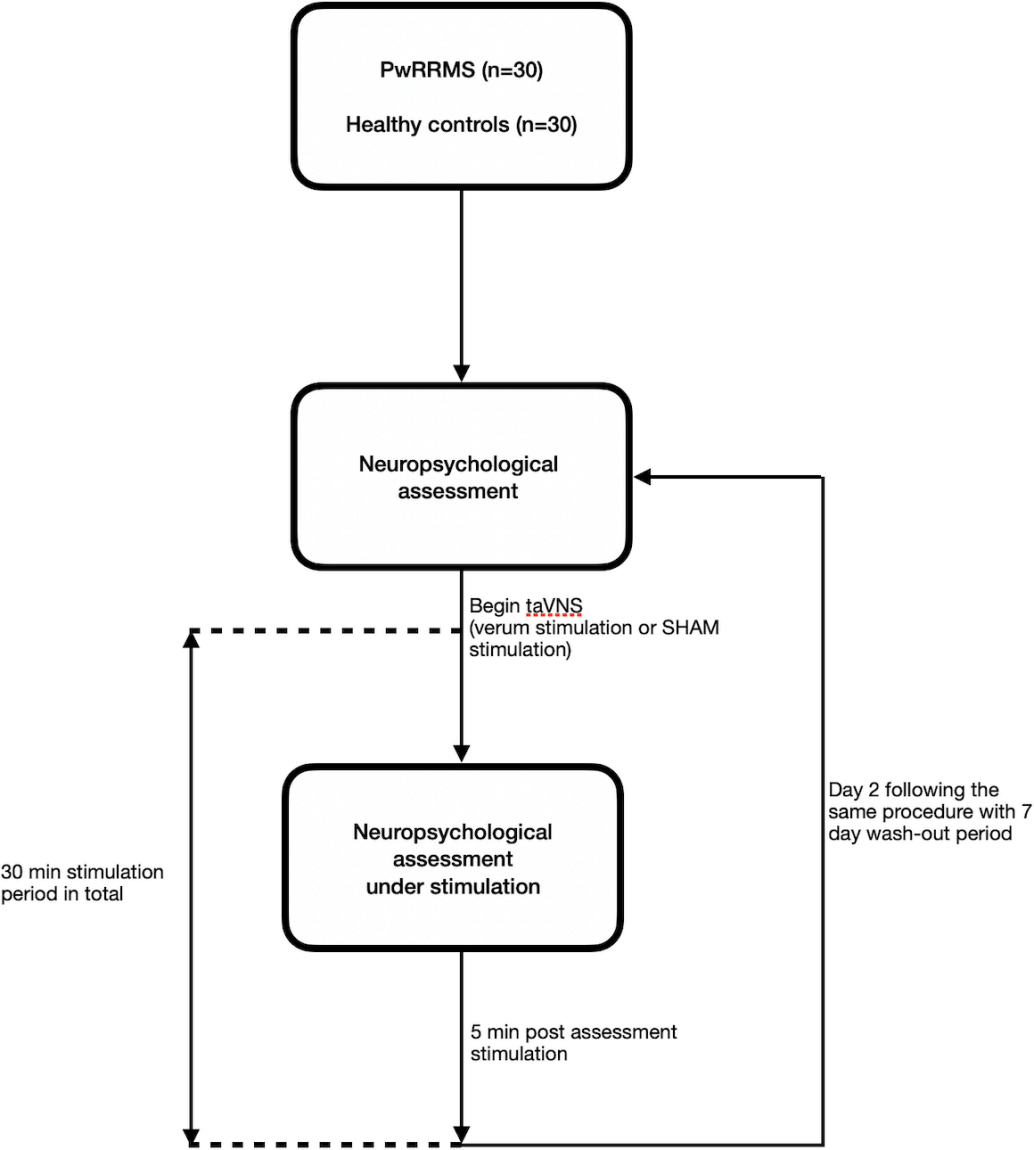
Study schedule. PwMS, people with relapsing remitting multiple sclerosis, taVNS, transcutaneous auricular vagus nerve stimulation.

This study comprises two groups, PwRRMS (n=30) and healthy controls (n=30). Both groups will undergo the same study schedule (figure 1). Patients will be blinded for the procedure using a lottery procedure to decide whether the participants start with SHAM or verum stimulation.

#### Baseline assessment

At the beginning, a medical interview will be conducted in which the relevant epidemiological and demographical data, including age and sex, are collected. Following the interview, a neuropsychological examination will be carried out, consisting of SDMT, BDI-II, FSMC and EDSS (the latter only used in PwRRMS).

#### Intervention

After baseline assessment, consisting of neuropsychological assessment (duration: about 30 min), stimulation (SHAM stimulation or verum stimulation) will be applied for 15 min followed by a second neuropsychological assessment under stimulation. In the first 15 min of stimulation (SHAM stimulation or verum stimulation), the participant will sit relaxed in a chair without a specific task. When the second neuropsychological assessment is completed, taVNS will be applied for another 5 min without a specific task.

#### Follow-up and further investigations

There will be a wash-out period of at least 7 days to eliminate possible long-term effects of stimulation. The second assessment on study day 2 will follow the same protocol as study day 1.

### Outcomes

#### Primary

The primary outcome measurement will be the processing speed in PwRRMS (SHAM stimulation vs verum stimulation), operationalised by the SDMT accuracy (total number of correct answers in 90 s).

#### Secondary

Exploratory outcome measurements will be:

1. The processing speed in healthy controls in relation to the stimulation method (SHAM stimulation and verum stimulation), operationalised by the SDMT accuracy.
2. The difference in the change in processing speed (SHAM stimulation vs verum stimulation) between the groups (PwRRMS/controls).
3. The association between disease-related variables (disease severity, fatigue and depression) and the change in processing speed.

### Statistical analysis

#### Sample size

To the best of our knowledge, there is no study investigating the effect of taVNS on processing speed in PwRRMS. Based on other studies assessing the effect of taVNS on non-motor symptoms like attentional processes or working memory, we plan a sample size of 60 subjects (30 healthy controls and 30 PwRRMS).^33 49 50^

#### Data analysis

Statistical analysis will be conducted using SPSS Statistics V.25 (IBM SPSS Statistics for Mac, V.25.0. Armonk, NY: IBM Corp). With a total number of n=30 in each study arm, a normal distribution can be assumed. Based on this, normally distributed variables will be presented as mean±SD. Non-normally distributed variables will be presented as median and (IQR). A linear mixed model will be performed for the primary/secondary outcomes using ‘time’ (pre vs during), ‘stimulation’ (taVNS vs sham), ‘group’ (MS vs controls), ‘session order’ (Session 1 vs Session 2) and ‘control variables’ (age, fatigue, depression and severity) as fixed, ‘participant’ as random effects. A significance level of 5% will be used to test the hypotheses.

## Discussion

taVNS is an already established non-invasive treatment option for depression or epilepsy.^51^,^53^ Recent studies investigating the effect of taVNS on cognition in healthy subjects provide preliminary evidence for its potential to also improve cognitive functions (eg, improvement of executive functions and spatial working memory).^23 24^

Mechanistically, taVNS might mediate its effect on cognition via different neurotransmitter systems and thus be a promising treatment option for cognitive impairments.^14 21^ For example, taVNS leads to an activation of the NTS which, in turn, leads to a stimulation of the locus caeruleus (release of norepinephrine).^54 55^ The increased levels of norepinephrine have widespread effects on cerebral structures that are important for different cognitive functions (eg, enhanced synaptic plasticity in the hippocampus).^56^

Furthermore, fMRI data revealed an anatomical overlap between SDMT performance and areas that are activated through taVNS application.^35^ A neurophysiological activation of these structures (such as the cerebellum or parietal cortical areas) might lead to an alteration in SDMT performance in PwRRMS.

Based on these findings, we hypothesise that taVNS will significantly improve processing speed in PwRRMS as well as in healthy controls (primary). Moreover, we will assess whether the stimulation-induced effect is also modulated by additional disease variables like disease severity, fatigue or depression (secondary).

To the best of our knowledge, this is the first study investigating the effect of taVNS in non-motor symptoms in PwRRMS (processing speed). With this study, we will determine whether taVNS improves an important aspect of cognitive function, that is, processing speed.

As in any other study, our study protocol has several limitations. First, this is a single centre, SHAM controlled, single-blinded prospective cohort study with a limited sample size of 30 participants in each group. This reduces the external validity of the study and might lead to an insufficient power to detect smaller effect sizes. To reduce interindividual differences, a cross-over design will be used. Secondary SHAM stimulation will be performed at the participants’ earlobe. Neuroanatomically, the earlobe is innervated by the great auricular nerve and not by the vagal nerve.^8^ Further studies revealed that the earlobe might not be a complete neutral SHAM condition, as the earlobe might still have vagal innervation.^57^ Despite the fact that the earlobe stimulation might cause a bias, we use the earlobe-SHAM-stimulation as it is still the standard SHAM stimulation. As there are no previous studies investigating the effect of taVNS on non-motor symptoms in PwRRMS, the optimal stimulation parameters cannot be clearly defined. We therefore use stimulation parameters that were already used to assess non-motor symptoms in other diseases besides multiple sclerosis.

Clinically, taVNS might be used as a new, non-invasive, well-tolerated and safe therapeutic option.^5 7^ A novel therapeutic strategy could enhance treatment modalities for multiple sclerosis and possibly also expand therapeutic options for other neurological disorders. Scientifically, these results may lead to a better understanding of how taVNS affects non-motoric symptoms in PwRRMS.

## Ethics and dissemination

The study protocol was reviewed and approved by the local ethics committee of the University Medical Centre Greifswald (study reference number: BB137/24; study registration: DRKS00034912). The study will be conducted in accordance with the Professional Code of Conduct for Physicians of the Medical Association of Mecklenburg-Vorpommern and the Declaration of Helsinki (V.1996). All collected data will be stored and processed pseudonymously according to currently existing data protection guidelines and laws. Names, examination results and other confidential information of the study participants are subject to medical confidentiality. Third parties do not have access to the original documents. Appropriate exceptions regarding the verification of the conformity of the original data, for example, by the ethics committee, are excluded. The study participants will be informed about the study procedure and potential risks associated with participation in the study. All study participants gave their informed consent before the start of the study.

To ensure data security, data of the participants will be pseudonymised and recorded containing participants IDs. Data will be secured with a password, solely accessible for study staff. Following good scientific practice, data will be stored for at least 10 years. Study results will be disseminated through academic conferences as well as peer-reviewed publications.

## Supplementary material

10.1136/bmjopen-2025-103998 online supplemental file 1
