# Supplementary material for: Effect of non-invasive transcutaneous auricular vagus nerve stimulation (taVNS) on non-motor symptoms in multiple sclerosis: study protocol for a randomised, controlled trial
Source: BMJ Open. 2025 Nov 9;15(11):e103998. doi: 10.1136/bmjopen-2025-103998 (PMC12598993; doi:10.1136/bmjopen-2025-103998)
Supplement: online supplemental file 1 [file bmjopen-15-11-s001.pdf]

Einwilligungserklärung

Effekt der nichtinvasiven transkutanen aurikulären Vagusnervstimulation auf die Verarbeitungsgeschwindigkeit (processing speed) von Patienten mit Multipler Sklerose

Studienleiter: Dr. med Thorsten Herr
Klinik und Poliklinik für Neurologie
Universitätsmedizin Greifswald
e-Mail: thorsten.herr@med.uni-greifswald.de

Beteiligte Studienzentren: Universitätsmedizin Greifswald, Klinik für Neurologie

Ich bin in einem persönlichen Gespräch ausführlich und verständlich über Wesen, Bedeutung, Ablauf, Risiken und Tragweite der Studie aufgeklärt worden.

Ich habe eine Kopie der Patienteninformation und Einwilligungserklärung ausgehändigt bekommen, diese gelesen und verstanden.

Ich hatte die Gelegenheit, über die Durchführung der Studie zu sprechen. Alle meine Fragen wurden zufrieden stellend beantwortet.

Möglichkeit zur Dokumentation zusätzlicher Fragen seitens der Patientin/des Patienten oder sonstiger Aspekte des Aufklärungsgesprächs:

____

____

____

____

Ich hatte ausreichend Zeit, um meine Entscheidung zu überdenken und frei zu treffen.

Ich stimme zu ☐ , ich stimme nicht zu ☐ ,

dass meine Kontaktdaten (Name, Telefonnummer) an die Studienleitung (Dr. med. Thorsten Herr) der Universitätsmedizin Greifswald weitergegeben werden, damit ich kontaktiert werde, um weitere Daten zu meiner Krankheitsanamnese/-verlauf zu erheben.

Ich bin mir bewusst und einverstanden, dass ich für die Überlassung meiner Krankheitsdaten kein Entgelt erhalte. Ich bin mir zudem bewusst, keinerlei Ansprüche auf Vergütung, Tantieme oder sonstige Beteiligung an finanziellen Vorteilen und Gewinnen zu haben, die möglicherweise auf der Basis der Forschung mit meinen Daten erlangt werden.

Mir ist bekannt, dass ich jederzeit und ohne Angabe von Gründen meine Einwilligung zur Teilnahme an der Studie zurückziehen kann, ohne dass mir daraus Nachteile entstehen. In diesem Fall werden alle studienbezogenen Daten gelöscht. Der Widerruf ist an die Studienleitung zu richten:

Dr. med Thorsten Herr

Klinik und Poliklinik für Neurologie

Universitätsmedizin Greifswald

e-Mail: thorsten.herr@med.uni-greifswald.de

…………………………………………………………………………………………………

Name des Patienten/der Patientin in Druckbuchstaben

…………………………………………………………………………………………………

Datum, Unterschrift

…………………………………………………………………………………………………

Name des aufklärenden Arztes / der aufklärenden Ärztin

…………………………………………………………………………………………………

Datum, Unterschrift

Sehr geehrte Patient*innen, sehr geehrte Proband*innen

wir bedanken uns für Ihr Interesse an der oben genannten Studie der Universitätsmedizin Greifswald.

Bei dieser Studie handelt es sich um eine wissenschaftliche Arbeit, die den Einfluss der sogenannten nichtinvasiven, transaurikulären Vagusnervstimulation auf unterschiedliche Symptome der Multiplen Sklerose untersucht. Dabei werden die Daten mit einer gesunden Kontrollgruppe verglichen.

Im folgenden werden Sie über die geplante Studie informiert.

1. Warum wird diese Studie durchgeführt?

Ziel dieser Studie ist es, den Einfluss des sogenannten Nervus vagus auf unterschiedliche Symptome der Multiplen Sklerose (MS) zu untersuchen. Hierzu zählen vorrangig die Verarbeitungsgeschwindigkeit, aber auch Symptome wie die Fatigue. Der Vagusnerv ist ein Hirnnerv, der auf viele Prozesse des unbewussten, sogenannten autonomen Nervensystems Einfluss hat. Daneben ist bekannt, dass eine Vagusnervstimulation auch auf allgemeine psychische Prozesse wie zum Beispiel Aufmerksamkeit wirkt. Wir wollen nun untersuchen, inwiefern sich die oben genannten Symptome nach einer Stimulation des Nervus vagus verändern.

Dazu werden wir den Vagusnerv mit Hilfe eines Stimulators, der am Ohr angebracht wird, stimulieren.

2. Wie ist der Ablauf der Studie und was muss ich bei der Teilnahme beachten?

Sie werden gebeten, insgesamt zwei Mal im Rahmen der Studie untersucht zu werden. Eine Untersuchung besteht jeweils aus einer neuropsychologischen Untersuchung (bestehend aus Unterschiedlichen Fragebögen sowie Aufgaben), gefolgt von einer transkutanen aurikulären Vagusnervstimulation (taVNS). Anschließend erfolgt eine erneute neuropsychologische Testung.

Nach Durchführung der neuropsychologischen Testung erfolgt eine taVNS-Untersuchung. Hierbei wird der Vagusnerv stimuliert. Dazu wird ein Stimulator verwendet, der am Ohr angebracht wird und Stromimpulse einer sehr geringen Intensität aussendet. In einem ersten Schritt wird getestet, wo Ihre individuelle Stimulationsschwelle liegt. Dabei wird die Intensität der Stromimpulse langsam immer weiter gesteigert. Die Stimulation an den zwei Untersuchungsterminen erfolgt dann mit gleicher Stimulationsstärke über jeweils 30 Minuten.

Nach der Stimulation erfolgt wie oben beschrieben, eine erneute neuropsychologische Testung, in welcher dann die Verarbeitungsgeschwindigkeit gemessen wird.

3. Welchen persönlichen Nutzen habe ich von der Teilnahme an dieser Studie?

Sie werden durch die Teilnahme an dieser Studie möglicherweise keinen persönlichen Gesundheitsnutzen haben. Die Ergebnisse der Studie könne aber möglicherweise dazu beitragen, die Vagusnervstimulation besser zu verstehen und gegebenenfalls diese als zusätzliche Therapieoption bei der Behandlung unterschiedlicher Symptome der Multiplen Sklerose zu etablieren.

4. Welche Risiken sind mit der Teilnahme an der Studie verbunden?

Ein Risiko bei Teilnahme an dieser Studie besteht nicht. Die Vagusnervstimulation ist bereits lange bekannt und wird bei Gesunden und Patienten in unterschiedlichen Krankheitsbildern eingesetzt.

5. Entstehen für mich Kosten durch die Teilnahme an der klinischen Studie? Erhalte ich eine Aufwandsentschädigung?

Durch Ihre Teilnahme an dieser Studie entstehen Ihnen keine zusätzlichen Kosten.

6. Bin ich während der klinischen Studie versichert?

Während der gesamten Studie, inklusive auf dem Weg zur Studie und zurück haben Sie einen vollumfänglichen Versicherungsschutz.

7. Wer entscheidet, ob ich aus der klinischen Studie ausscheide?

Sie könne jederzeit, auch ohne Angabe von Gründen, Ihre Teilnahme beenden, ohne dass Ihnen dadurch irgendwelche Nachteile bei Ihrer medizinischen Behandlung entstehen. In diesem Fall werden die erhobenen Daten gelöscht.

8. Was geschieht mit meinen Daten?

Während der klinischen Studie werden persönliche Informationen von Ihnen erhoben. Die für die Studie wichtigen Daten werden in pseudonymisierter Form gespeichert und ausgewertet.

Pseudonymisiert bedeutet, dass keine Angaben von Namen oder Initialen verwendet werden, sonder nur ein Nummerncode. Die Daten sind gegen unbefugten Zugriff gesichert. Eine Entschlüsselung erfolgt nur unter den vom Gesetz vorgeschriebenen Voraussetzungen.

9. An wen wende ich mich bei weiteren Fragen?

Sie haben stets die Gelegenheit zu weiteren Beratungsgesprächen mit dem auf Seite 1 genannten oder einem anderen Prüfarzt. Die Untersuchung findet unter der wissenschaftlichen Leitung von Herrn Dr. med Thorsten Herr statt.

Datenschutzerklärung:

Mir ist bekannt, dass bei dieser Studie personenbezogene Daten über mich erhoben, gespeichert und ausgewertet werden sollen. Die Verwendung der Daten erfolgt nach gesetzlichen Bestimmungen und setzt vor der Teilnahme an der Studie folgende freiwillig abgegebene Einwilligungserklärung voraus, das heißt, ohne die nachfolgende Einwilligung kann ich nicht an der Studie teilnehmen.

1. Ich erkläre mich weiterhin einverstanden, dass im Rahmen dieser Studie personenbezogene Daten über mich erhoben und in Papierform sowie auf elektronischen Datenträgern in pseudonymisierter Form von der Universitätsmedizin Greifswald gespeichert werden.

Die für die Datenverarbeitung verantwortliche Person der Studie ist Dr. med. T. Herr, Klinik und Poliklinik für Neurologie, Sauerbruchstraße, 17487 Greifswald. e-Mail: Thorsten.herr@med.uni-greifswald.de

Datenschutzbeauftragter der für die Datenverarbeitung der Studie verantwortliche Person ist Prof. Ulf Glende, Walther-Rathenau-Str. 49, 17475 Greifswald. Email: datenschutz-umg@med.uni-greifswald.de

2. Ich bin auf das Beschwerderecht bei einer Datenschutzaufsichtsbehörde hingewiesen worden. Die für die Studie zuständige Datenschutz-Aufsichtsbehörde lautet: Der Landesbeauftragte für Datenschutz und Informationsfreiheit Mecklenburg-Vorpommern, Werderstraße 74a, 19055 Schwerin, Email: info@datenschutz-mv.de

3. Ich bin darüber aufgeklärt worden, dass ich die Teilnahme an der Studie jederzeit und ohne Angabe von Gründen gegenüber den Prüfärzten der Klinik für Neurologie der Universitätsmedizin Greifswald widerrufen kann. Im Falle des Widerrufs werden meine Daten gelöscht.

4. Ich erkläre mich damit einverstanden, dass meine Daten nach Beendigung oder Abbruch der Studie mindestens zehn Jahre aufbewahrt werden. Danach werden meine personenbezogenen Daten gelöscht, soweit nicht gesetzliche Aufbewahrungsfristen entgegenstehen.

5. Schließlich erkläre ich auch mein Einverständnis für die wissenschaftliche Veröffentlichung der Forschungsergebnisse unter Beachtung der datenschutzrechtlichen Bestimmungen.

6. Ich habe vom datenschutzrechtlichen Einsichts- und Korrekturrecht bezüglich meiner personenbezogenen Daten Kenntnis genommen. Ich bin auf das Recht hingewiesen worden, Auskunft (einschließlich kostenfreier Überlassung einer Kopie) über mich betreffende personenbezogene Daten zu erhalten sowie ggf. deren Berichtigung oder Löschung zu verlangen.

……………………………………………………………………………………………………

Name des Patienten/der Patientin in Druckbuchstaben

……

Datum, Unterschrift

……

Name des aufklärenden Arztes / der aufklärenden Ärztin

……

Datum, Unterschrift
