# Supplementary material for: Effect of non-invasive transcutaneous auricular vagus nerve stimulation (taVNS) on non-motor symptoms in multiple sclerosis: study protocol for a randomised, controlled trial
Source: BMJ Open. 2025 Nov 9;15(11):e103998. doi: 10.1136/bmjopen-2025-103998 (PMC12598993; doi:10.1136/bmjopen-2025-103998)
Supplement: Supplementary file 2 [file bmjopen-2025-103998.reviewer_comments.pdf]

PEER REVIEW HISTORY

BMJ Open publishes all reviews undertaken for accepted manuscripts. Reviewers are asked to complete a checklist review form and are provided with free text boxes to elaborate on their assessment. These free text comments are reproduced below.

ARTICLE DETAILS

Title (Provisional)

Effect of non-invasive transcutaneous auricular vagus nerve stimulation (taVNS) on non-motor symptoms in multiple sclerosis - Study protocol for a randomized, controlled trial

Authors

Herr, Thorsten; Gamain, Julie; Strauss, S.; Szeska, C.; Weymar, Mathias; Flöel, Agnes; Penner, Iris-Katharina; Grothe, M.

VERSION 1 - REVIEW

Reviewer 1

Name Mortimer, Gierthmuehlen

Affiliation University Medical Center Knappschaftskrankenhaus Bochum, Neurosurgery

Date 20-Jun-2025

COI None

Page 1 Line 40: translate in english

Page 3, line 38: first of may, isn't it better to write 1st May?

Page 3, line 55: "any" cardiovascular disease is an exclusion criteria? Not only s/p infarction, bradykardia, etc.? Exclusion criteria are pretty wide...

Page 4, line 18: the copyright holder Hogrefe for BDI needs to be cited correctly

Page 4, line 40: which tVNS-System is used? tVNS-R or L? The R-System does not have a CE-mark as a medical device

Page 4, line 59: the paradigm is usually not 30s on / 30s off – it is 28s on / 32s off

Page 5, line 2: what time of day?

Page 5, line 5: will participants stay in their respective group? Or will they switch from verum to sham and vice versa?

Discussion is a bit short – legal aspects are lacking, critical discussion of sham-procedure (this kind of sham is often used, but also being criticized as the earlobe might still have some vagal innveration) should be added. Sample size calculation in my eyes a bit short.

Please make sure to have the copyright to use the respective questionnaires:

Symbol Digit Modality Test : Western Psychological Services (WPS)

BDI-2 : Hogrefe Verlag

**Reviewer**	**2**
**Name**	**León-Ruiz, Moisés**
**Affiliation**	**La Paz University Hospital, Department of Neurology**
**Date**	**06-Aug-2025**
**COI**	**None**

A very interesting article about Effect of non-invasive transcutaneous auricular vagus nerve stimulation (taVNS) on non-motor symptoms in multiple sclerosis - Study protocol for a randomized, controlled trial. Congratulations

---

VERSION 1 - AUTHOR RESPONSE

Reviewer: 1 Dr. Gierthmuehlen Mortimer, University Medical Center Knappschaftskrankenhaus Bochum Comments to the Author:

* Page 1 Line 40: translate in english
> Thank you for the comment. We are sorry for the mistake. Strengths and limitations were completely revised.

* Page 3, line 38: first of may, isn't it better to write 1st May?
> We added „1st May" instead of the previous date. Furthermore English language editing was improved by a native speaking researcher.

* Page 3, line 55: "any" cardiovascular disease is an exclusion criteria? Not only s/p infarction, bradykardia, etc.? Exclusion criteria are pretty wide...
> Thank you for this relevant comment. The exclusion criteria were pretty wide, so we added concrete exclusion criteria with corresponding examples.

* Page 4, line 18: the copyright holder Hogrefe for BDI needs to be cited correctly
> We added the correct references for the Beck Depression Inventory.

* Page 4, line 40: which tVNS-System is used? tVNS-R or L? The R-System does not have a CE-mark as a medical device
> In our study we use the taVNS-R device to modulate the different stimulation parameters. Although it does not have a CE certification, we prefer the taVNS-R device because of the possibility to alter parameters specifically for different studies.

* Page 4, line 59: the paradigm is usually not 30s on / 30s off – it is 28s on / 32s off
> Thank you for the comment. Based on previous taVNS studies using the device from tVNS technologies (e.g., Farmer et al. 2021) and from our own research we are mainly using the default setting which includes alternating stimulation of 30s on and 30s off (see also Ventura-Bort et al 2025, Szeska et al 2025).

* Page 5, line 2: what time of day?
> Thank you for the comment, we specified the time of day accordingly. In our study, we want to control for circadian influences. Hence, the time of assessments will be kept constant within participants (i.e., if the first assessment will be at around 10am, the second assessment will also take place at around 10am). Following sentence was added: „In order to control for circadian influences within subjects, stimulation will be applied at a constant time for each participant (when the participant will be assessed in the morning, the second assessment will also be performed in the morning)."

* Page 5, line 5: will participants stay in their respective group? Or will they switch from verum to sham and vice versa?
> We are sorry for the unclear wording in the protocol. We are using a cross-over design. Thus, participants will switch between SHAM and verum stimulation and vice versa. We therefore added following sentence: „In summary, every participant will switch from verum to sham and vice versa."

* Discussion is a bit short – legal aspects are lacking, critical discussion of sham-procedure (this kind of sham is often used, but also being criticized as the earlobe might still have some vagal innveration) should be added. Sample size calculation in my eyes a bit short.
> We revised the discussion and added a critical discussion of SHAM stimulation. Furthermore, we added specific limitations of the planned study as well as a theoretically possible effect of taVNS on processing speed.

* Please make sure to have the copyright to use the respective questionnaires: Symbol Digit Modality Test : Western Psychological Services (WPS) BDI-2 : Hogrefe Verlag
> We are using the copyright version of the tasks when starting the study. The tests are already established in our clinic and the corresponding copyrights are available.

Reviewer: 2 Dr. Moisés León-Ruiz, La Paz University Hospital Comments to the Author: A very interesting article about Effect of non-invasive transcutaneous auricular vagus nerve stimulation (taVNS) on non-motor symptoms in multiple sclerosis - Study protocol for a randomized, controlled trial. Congratulations
> Thank you for this comment. We highly appreciate your opinion on our research protocol and are glad to hear that the article leads to interest.

VERSION 2 - REVIEW

Reviewer **1**

Name **Mortimer, Gierthmuehlen**

Affiliation **University Medical Center Knappschaftskrankenhaus Bochum, Neurosurgery**

**Date**	**18-Sep-2025**
**COI**	

Well done!
